# The Steiner cycle and path cover problem on interval graphs

**DOI:** 10.1007/s10878-021-00757-7

**Published:** 2021-05-27

**Authors:** Ante Ćustić, Stefan Lendl

**Affiliations:** 1 Department of Mathematics, Simon Fraser University Surrey, 250-13450 102nd AV, Surrey, British Columbia V3T 0A3 Canada; 2 Institute of Discrete Mathematics, Graz University of Technology, Steyrergasse 30, 8010 Graz, Austria; 3 Department of Operations and Information Systems, University of Graz, Graz, Austria

**Keywords:** Interval graphs, Steiner cycle, Hamiltonian cycle, Linear time

## Abstract

The Steiner path problem is a common generalization of the Steiner tree and the Hamiltonian path problem, in which we have to decide if for a given graph there exists a path visiting a fixed set of terminals. In the Steiner cycle problem we look for a cycle visiting all terminals instead of a path. The Steiner path cover problem is an optimization variant of the Steiner path problem generalizing the path cover problem, in which one has to cover all terminals with a minimum number of paths. We study those problems for the special class of interval graphs. We present linear time algorithms for both the Steiner path cover problem and the Steiner cycle problem on interval graphs given as endpoint sorted lists. The main contribution is a lemma showing that backward steps to non-Steiner intervals are never necessary. Furthermore, we show how to integrate this modification to the deferred-query technique of Chang et al. to obtain the linear running times.

## Introduction

In this paper we investigate the *Steiner cycle* and *Steiner path* problem on interval graphs. To our knowledge, these problems have not been studied for this specific graph class. However, the *Hamiltonian cycle* and *Hamiltonian path* problem, which are special cases of the Steiner variants, are extensively studied for interval graphs and can be solved in linear time, if the intervals are given as an endpoint sorted list (Hung and Chang 2011; Keil 1985; Arikati and Rangan 1990; Manacher et al. 1990).

Steiner path problems have already been studied for other special graph classes, like trees (Moharana et al. 2013) and directed co-graphs (Gurski et al. 2020a, b). For the feasibility variant of the Steiner cycle problem, the so-called *k*-*cycle* problem an exact FPT algorithm has been obtained (Wahlström 2013). Also, the Steiner cycle polytope has been studied (Salazar-Gonzalez 2003) and approximation algorithms were studied (Steinová 2010). Steiner cycles have applications in the optimal design of reliable telecommunication and transportation networks (Steiglitz et al. 1969).

In this work we generalize the algorithms of Manacher et al. (1990) to the *Steiner setting* and obtain first linear time algorithms for the *Steiner path cover* and *Steiner cycle* problem on interval graphs. To obtain our results we extend the tools introduced in Hung and Chang (2011), and prove an elegant skipping lemma for the Steiner setting.

## Definitions and preliminary results

As usual, we denote by $G=(V,E)$ a graph, where *V* is the set of vertices and *E* is a set of pairs of vertices (two element sets of vertices), the edges. Given an interval $i = [x, y]$ we denote the left endpoint *x* by ${\text {l}}(i) = x$ and the right endpoint *y* by ${\text {r}}(i) = y$. Let $I = (i_{1}, i_{2}, \dots , i_{n})$ be a list or set of *n* intervals. We denote by *G*(*I*) the interval graph of *I*. The vertices of *G*(*I*) correspond to the intervals of *I*. Two intervals $i, i' \in I$ are connected by an edge in *G*(*I*) if $i \ne i'$ and $i \cap i' \ne \emptyset$.

For an arbitrary graph $G = (V, E)$ a list of vertices $P = (v_{1}, v_{2}, \dots , v_{l})$ is a (simple) path if those vertices are pairwise distinct and for each $j=1,2,\dots ,l-1$ it holds that $\{v_{j}, v_{j+1}\} \in E$. The start of *P* is denoted by ${\text {start}}(P) = v_{1}$ and the end of *P* is denoted by ${\text {end}}(P) = v_{l}$. We define ${\text {rev}}(P)$ as the reverse path $(v_{l}, v_{l-1}, \dots , v_{1})$ of *P*. If in addition $\{v_{l}, v_{1}\} \in E$ holds, then we call *P* a (simple) cycle. For ease of writing we sometimes abuse notation and consider *P* as a set instead of a list, to allow for the use of set operations. Given two paths *P* and *Q* and a vertex *v* we also write (*P*, *Q*) for the concatenation of *P* and *Q*, and (*P*, *v*) for the concatenation of *P* and *v*.

Given a set $S \subseteq V$, a *Steiner cycle* is a cycle *C* in *G* such that $S \subseteq C$. A *Steiner path cover* of *G* is a set $\{P_{1}, P_{2}, \dots , P_{k}\}$ of pairwise disjoint paths in *G* such that $S \subseteq \bigcup _{j=1}^{k} P_{j}$. The *Steiner path cover number* $\pi _{S}(G)$ is the the minimum cardinality of a Steiner path cover of *G*. If $\pi _{S}(G) = 1$ we say that *G* has a *Steiner path*.

A set $C \subseteq V$ is called a *cutset* of *G* if $G-C$ is disconnected. A set of vertices $T \subseteq V$ is called an island with respect to *C*, if *T* is not adjacent to any vertex in $V\setminus (C \cup T)$. *T* is called an *S*-island with respect to *C*, if *T* is an island with respect to *C* and $S \cap T \ne \emptyset$.

The following two results for general simple graphs are generalizations of two observations by Hung and Chang (2011).

### Proposition 2.1

Let *C* be a cutset of *G* and $g_{S}$ the number of connected components *K* in $G-C$ such that $K \cap S \ne \emptyset$. Then, $\pi _{S}(G) \ge g_{S} - |C|$.

### Proof

Let $(P_{1}, P_{2}, \dots , P_{k})$ be a Steiner path cover of *G*. For every $P_{j}$ let $g_{j}$ be the number of components *K* of $G-C$ with $K \cap S \ne \emptyset$ and $K \cap P_{j} \ne \emptyset$. The $P_{j}$ must use at least $g_{j} - 1$ distinct vertices from *C* to reconnect itself from those different components of $G-C$, i.e., $|P_{j} \cap C| \ge g_{j} - 1$. Now, since paths of a path cover are vertex disjoint, we have that $|C| \ge \sum _{j=1}^{k} (g_{j} - 1)$. Finally, from the fact that $\sum _{j=1}^{k} g_{j} \ge g_{S}$, we get $k \ge g_{S} - |C|$, which proves our claim. $\square$

### Proposition 2.2

Let *C* be a cutset of *G* and $g_{S}$ the number of connected components *K* in $G-C$ such that $K \cap S \ne \emptyset$. If $g_{S} > |C|$, then *G* has no Steiner cycle.

### Proof

A Steiner cycle needs to connect all components *K* of $G-C$ with $K \cap S \ne \emptyset$. For each such connection a distinct vertex from *C* has to be used. Since it is a cycle, it has to be closed, hence $g_{S}$ such connections are necessary. This implies that if $g_{S} > |C|$ no Steiner cycle can exist. $\square$

We use these results to solve the *Steiner path cover* problem (see Sect. 3) and the *Steiner cycle* problem (see Sect. 4) on interval graphs efficiently. Throughout the paper we assume that |*S*| is known to the algorithms and queries $i \in S$ can be performed in *O*(1) time.

## The Steiner path cover problem

We show that the basic greedy principle, that is at the core of efficient algorithms for the *path cover* problem on interval graphs, can be generalized by the introduction of *neglectable intervals*. But first we explain the basic greedy principle to find paths in interval graphs that was introduced independently by Manacher et al. (1990) and Arikati and Rangan (1990).

Given an endpoint sorted list of intervals we number those intervals as $i_{1}, i_{2}, \dots , i_{n}$ in increasing order with respect to their right endpoint, hence $r(i_{j}) < r(i_{j+1})$ for all $j = 1,2,\dots ,n-1$. (Such a numbering can be easily obtained in *O*(*n*) time using a list which is sorted by endpoints of the intervals and is assumed for the rest of this paper. W.l.o.g. we can assume that $r(i_{j}) \ne r(i_{k})$ for $i \ne k$. ) The algorithm iteratively constructs a path *P*. It starts with the path $P := (i_{1})$ containing only the first interval. Then in each iteration it extends *P* by the unique neighbor of ${\text {end}}(P)$ which is not already contained in *P* with minimum right endpoint. If no such extension is possible the algorithm terminates with the current path *P* as an output. We denote this algorithm by $\mathbf {GP}$ and the path *P* obtained by this algorithm by $\mathbf {GP}(I)$.

For a path $P = \mathbf {GP}(I) = (v_{1}, v_{2}, \dots , v_{l})$ obtained by executing the algorithm on an interval graph *G*(*I*), we define *L*(*P*) as the set of intervals of *P* that exceed beyond the right endpoint of ${\text {end}}(P)$, i.e. $L(P) = \{ v \in P :{\text {r}}(v) > {\text {r}}({\text {end}}(P)) \}$. Now we can recursively define *C*(*P*), the set of *covers* of the path *P*, as follows. If $L(P) = \emptyset$, we set $C(P) = \emptyset$. Otherwise, let *j* be the maximum index such that $v_{j} \in L(P)$. We set $C(P) = \{v_{j}\} \cup C(P')$ for $P' = (v_{1}, v_{2}, \dots , v_{j-1})$.

For $C(P) = \{c_{1}, c_{2}, \dots ,c_{k} \}$ and $P = (P_{0}, c_{1}, P_{1}, c_{2}, \dots , c_{k}, P_{k})$, Manacher et al. (1990) proved that for each $j=0,1,\dots , k$ it holds that $P_{j}$ is an island with respect to *C*(*P*) and if $I\setminus P \ne \emptyset$ also $I \setminus P$ is an island with respect to *C*(*P*). Such a representation of *P* we call a *decomposition into covers and islands*.

The following property directly follow from the definition of a decomposition into covers and islands and are already essential in the proofs in Manacher et al. (1990).

### Proposition 3.1

Let $P = \mathbf {GP}(I) = (v_{1}, v_{2}, \dots , v_{l})$. If $P = (P_{0}, c_{1}, P_{1}, c_{2}, \dots , c_{k}, P_{k})$ is a decomposition into covers and islands, then it holds that $L(P_{j}) = \emptyset$ for each $j=0,1,\dots ,k$.

### Proof

We prove this fact by induction on the number of covers *k*. If $k=0$ we have that $P = (P_0)$ and $C(P) = C(P_0) = \emptyset$. By defintion of $C(P_0)$ it holds that also $L(P) = L(P_0) = \emptyset$. Otherwise, consider $P = (v_{1}, v_{2}, \dots , v_{l})$ with $(P_0, c_1, P_1, c_2, \dots , c_k, P_k)$ for $k \ge 1$ its decomposition into covers and islands. By the definition of *C*(*P*) it holds that $c_k = v_j$ for *j* the maximum index such that $v_j \in L(P)$. Hence, there exists no $j' > j$ such that $r(v_j') > {\text {end}}(P) = {\text {end}}(P_k)$. This implies that $L(P_k) = \emptyset$. For all $i < k$ it follows by induction that $L(P_i) = \emptyset$. $\square$

**Fig. 1:**
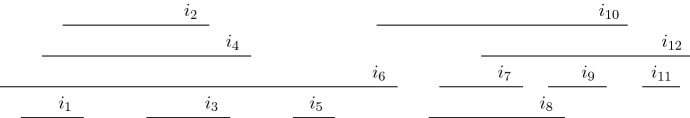
An interval model *I* of twelve endpoint-sorted intervals (Hung and Chang 2011)

To illustrate the notions introduced above, consider the intervals in Fig. 1 given as a right endpoint-sorted list $I = (i_1, i_2,\ldots , i_{12})$. Algorithm $\mathbf {GP}$ starts by setting $P=(i_1)$. Neighbors of $i_1$ are $\{ i_2, i_4, i_6\}$, and since ${\text {r}}(i_2) < \min \{{\text {r}}(i_4), {\text {r}}(i_6)\}$ we extend *P* by $i_2$, i.e. $P = (i_1, i_2)$. Among neighbors of $i_2$ that are not already in *P*, $i_3$ has the smallest right endpoint, so *P* is extended to $P=(i_1, i_2, i_3)$. Next candidates for the extension are $\{i_4, i_6 \}$ among which we chose $i_4$, i.e. $P=(i_1, i_2, i_3, i_4)$. Next, the only possible extension is by $i_6$, hence $P=(i_1, i_2, i_3, i_4, i_6)$. Among the next candidates for extension $\{i_5, i_{10} \}$, interval $i_5$ is chosen. At this point the algorithm terminates and outputs $P = (i_1, i_2, i_3, i_4, i_6, i_5)$, since there is no neighbor of $i_5$ that is not already in *P*.

Now we find a decomposition into covers and islands of *P*. Since ${\text {r}}(i_6) > {\text {r}}({\text {end}}(P)= i_5)$, we have that $L(P) = \{i_6\}$, and $C(P) = \{i_6\} \cup C(P'=(i_1, i_2, i_3, i_4))$. $L(P')$ is the empty set, so the decomposition process is over and we have that the decomposition into covers and islands of *P* is given by $C(P) = \{i_6\}$ and $P=(P_0, i_6, P_1)$, where $P_0= (i_1, i_2, i_3, i_4)$ and $P_1= (i_5)$. Note that $P_0$, $P_1$ and $I \setminus P$ are islands with respect to $C(P)= \{i_6\}$. Furthermore, note that our decomposition satisfies the properties in Proposition 3.1.

Given the fact that in the Steiner variant of the problem only the intervals in *S* have to be visited, we introduce *neglectable intervals*. Let *P* be the current path at any point of the greedy algorithm and $v'$ be the next extension. We call $v'$ *neglectable* with respect to ${\text {end}}(P)$, if $v' \notin S$ and ${\text {r}}(v') < {\text {r}}({\text {end}}(P))$, i.e. ${\text {end}}(P) \in L((P, v'))$. We modify the algorithm $\mathbf {GP}$, such that it skips neglectable intervals with respect to the end of the current path. Analogously to $\mathbf {GP}$ this modification is denoted by $\mathbf {GP}_{S}$. Additional two distinctions of $\mathbf {GP}_S$ are that it starts with the interval (with the smallest ${\text {r}}(v)$) that is in *S*, and it terminates as soon as there are no more uncovered intervals in *S*. We denote by $N_{v}$ the set of intervals that are not contained in $\mathbf {GP}_{S}(I)$ because they are neglectable with respect to *v* for some path *P* during the execution of $\mathbf {GP}_{S}$, where $v = {\text {end}}(P)$. Let *N* be the set of all such neglectable intervals obtained during the entire run of $\mathbf {GP}_{S}$.

Now we present a lemma which is our main tool for elegantly proving our main results.

### Lemma 3.2

Let $P = \mathbf {GP}_{S}(I)$ be the path obtained by $\mathbf {GP}_{S}$ for a given list of intervals *I*, let $P = (P_{0}, c_{1}, P_{1}, c_{2}, \dots , P_{k-1}, c_{k}, P_{k})$ be its decomposition into covers and islands in $G(I\setminus N)$, and let $C(P) = \{c_{1}, c_{2}, \dots , c_{k}\}$. Then it holds for all $j=0,1,\dots ,k$ that $P_{j} \cap S \ne \emptyset$, i.e. $P_{j}$ is an *S*-island with respect to *C*(*P*) in $G(I\setminus N)$. It even holds that $P_{j} \cup N_{c_{j}}$ contains at least one *S*-island with respect to *C*(*P*) in *G*(*I*).

### Proof

It is easy to see that this decomposition into covers and islands exists, since if $P = \mathbf {GP}_{S}(I)$ it follows by construction that $P = \mathbf {GP}(I\setminus N)$.

The fact that $P_{j}$ is an *S*-island with respect to *C*(*P*) in $G(I\setminus N)$ is a trivial consequence of the decomposition into covers and islands and the definition of $\mathbf {GP}_{S}$. Since $c_{j}$ is used before every interval in $N_{c_{j}}$ we have that the left endpoint of every interval in $N_{c_{j}}$ is larger than the left endpoint of $c_{j}$. The right endpoints of each of the intervals in $N_{c_{j}}$ is smaller than the right endpoint of $c_{j}$ by definition of neglected intervals. But this directly implies that *C*(*P*) separates also $N_{c_{j}}$ from the rest of *G*(*I*), except for possibly $P_{j}$. $\square$

Now we can obtain an easy procedure to solve the Steiner path cover problem on interval graphs. We start with ${\mathcal {P}} = \emptyset$ and apply the algorithm $\mathbf {GP}_{S}$. After termination let $P = \mathbf {GP}_{S}(I)$. We add *P* to our partial solution ${\mathcal {P}}$ and find the smallest index *j* such that $i_{j} \in S$ and $i_{j}$ is not in any path currently contained in ${\mathcal {P}}$. Then we apply $\mathbf {GP}_{S}$ again to the list of intervals $i_{j}, i_{j+1}, \dots , i_{n}$. We iterate like this until all intervals in *S* are covered by one of the paths in ${\mathcal {P}}$. The algorithm terminates with the Steiner path cover ${\mathcal {P}}$ as its output.

### Theorem 3.3

The Steiner path cover obtained by iterated application of $\mathbf {GP}_{S}$ is optimal.

### Proof

Let $P_{1}, P_{2}, \dots , P_{l}$ be the paths obtained by the algorithm and $C' = \bigcup _{j=1}^{l} C(P_{j})$ be the union of all the covers in the decomposition into covers and islands of each path. Then, by repeated application of Lemma 3.2 we obtain that there are $l+|C'|$ *S*-islands with respect to $C'$ in *G*(*I*). By Proposition 2.1 we then know that $\pi _{S}(G(I)) \ge l$, so our solution is an optimal Steiner path cover. $\square$

To illustrate our algorithm for the Steiner path cover problem we again consider the example in Fig. 1. In the case when $S= I$, i.e., all intervals need to be covered, our algorithm runs $\mathbf {GP}_S(I)$ which outputs $P'=(i_1, i_2, i_3, i_6, i_5)$, and then it runs $\mathbf {GP}_S(I\setminus P')$ which outputs $P''=(i_7, i_8, i_9, i_{10}, i_{12}, i_{11})$, and the algorithm terminates. Therefore, for $S=I$ we have that $\pi _S(I)=2$. Now lets say that $S=\{i_2, i_4, i_6, i_8, i_{10}, i_{12}\}$. $\mathbf {GP}_S(I)$ starts with the element of *S* with the smallest right endpoint which is $i_2$. Then it extends the path with $i_3$, $i_4$ and then $i_6$. After that, the algorithm neglects $i_5$ since ${\text {r}}(i_5) < {\text {r}}(i_6)$ and $i_5 \notin S$. Next, the path is extended by $i_{10}$, then $i_7$ is neglected, but $i_8$ is added to the path (since $i_8 \in S$). Then the path is extended by $i_9$ and finally by $i_{12}$. Interval $i_{11}$ is neglected. The output of the algorithm is the path $P=(i_2, i_3, i_4, i_6, i_{10}, i_8, i_9, i_{12})$, so $\pi _S(I)= 1$. Note that the key factor that allowed us to cover the set *S* with only one path is the fact that we could neglect $i_5$.

By using the deferred-query approach by Chang et al. (1999) this algorithm can be implemented in *O*(*n*) time.

### Theorem 3.4

The iterated application of $\mathbf {GP}_{S}$ can be implemented in *O*(*n*) time, using the deferred-query approach.

### Proof

Firstly, we give a high-level explanation of how to implement $\mathbf {GP}_{S}$ using the deferred-query technique. Afterwards we argue how the modifications can still be implemented in linear time.

In the deferred-query approach the algorithm handles the intervals in the given right endpoint sorted order one after another. For each *j* where $i_{j-1} \cap i_{j} \ne \emptyset$ the algorithm can be executed as stated above, since in this case we have that ${\text {end}}(P) = i_{j-1}$ and we extend *P* with $i_{j}$. The main difference is, that when $i_{j-1} \cap i_{j} = \emptyset$ we still have to process $i_{j}$ instantly.

This is handled in the following way: The algorithm keeps at each time a collection of paths $P_{1}, \dots , P_{l}$ and a flag that indicates whether $P_{l}$ already contains an interval from *S*. (For all other paths it is an invariant of the algorithm that they always contain an interval from *S*). In the beginning we have that $l=1$ and $P_{1} = (i_{1})$, where we assume that $i_{1} \in S$. When handling $i_{j}$ we have the following case distinction.

- ${\text {end}}(P_{l}) \cap i_{j} \ne \emptyset$ and ${\text {end}}(P_{k}) \cap i_{j} = \emptyset$ for all $k < l$: in this case, if $P_{l}$ contains an interval from *S* we extend $P_{l}$ by $i_{j}$, hence $P_{l} := (P_{l}, i_{j})$. Otherwise we set $P_{l} := (i_{j})$.
- There is a $k<l$ such that ${\text {end}}(P_{k}) \cap i_{j} \ne \emptyset$: let *k* be minimum with this property. Then also ${\text {start}}(P_{k+1}) \cap i_{j} \ne \emptyset$ and hence we can connect $P_{k}$ and $P_{k+1}$ using $i_{j}$, hence the new collection of paths is $P_{1}, \dots , (P_{k}, i_{j}, P_{k+1}), \dots , P_{l}$. If $k+1 = l$ and $P_{l}$ does not contain an interval from *S* we instead set the new collection of paths to $P_{1}, \dots , (P_{l-1}, i_{j})$.
- ${\text {end}}(P_{l}) \cap i_{j} = \emptyset$: In this case if $P_{l}$ contains an interval from *S* we add a new path $P_{l+1} := (i_{j})$, hence the new collection of paths is $P_{1}, P_{2}, \dots , P_{l}, P_{l+1}$. Otherwise we replace $P_{l} := (i_{j})$.

It is now easy to see that after termination this algorithm obtains exactly the set $(P_{1}, \dots , P_{l})$ that is the output of iterating algorithm $\mathbf {GP}_{S}$. The main observation is that the intervals that are removed by the procedure above are either neglectable or are intervals not in *S* that are strictly between ${\text {r}}({\text {end}}(P_{k}))$ and ${\text {l}}({\text {start}}(P_{k+1}))$ for some $k=1,\dots ,l-1$.

It remains to show that interval $i_{j}$ can be handled in *O*(1) time. This follows directly by the implementation based on *static tree set union* shown in by Chang et al. (1999), since the only difference is that in each step we have to do a case distinction for whether the current $P_{l}$ contains an interval from *S*. The operations performed then correspond to operations already performed by the original algorithm, and removing the current last path $P_{l}$. This remove operation can obviously also be handled in constant time. $\square$

## The Steiner cycle problem

Next we generalize the algorithm of Manacher et al. (1990) to solve the Steiner cycle problem on interval graphs. We first run our algorithm for the Steiner path cover problem (see Sect. 3). If $\pi _{S} > 1$ we know that there cannot exist a Steiner cycle. Otherwise, let $P = (v_{1}, v_{2}, \dots , v_{l})$ be the obtained Steiner path in *G*(*I*).

Based on *P* we construct two paths *Q* and *R*. We start by setting $R := (v_{1})$ and $Q = (v_{2})$. Then, we iteratively process the intervals $v_{3}$ to $v_{l}$. If in the step of processing interval $v_{j}$ we have that ${\text {end}}(Q) = v_{j-1}$, we consider the following two cases. If $v_{j} \cap {\text {end}}(R) \ne \emptyset$, we extend *R* by $v_{j}$, i.e. $R = (R, v_{j})$. Otherwise, we extend *Q* by $v_{j}$, i.e. $Q = (Q, v_{j})$. If on the other hand in this step we have that ${\text {end}}(R) = v_{j-1}$ we analogously check if $v_{j} \cap {\text {end}}(Q) \ne \emptyset$. If this is the case we extend *Q* by $v_{j}$ and if not we extend *R* by $v_{j}$.

In the end of this process we try to connect *R* and ${\text {rev}}(Q)$ to a Steiner cycle. To achieve this we check if ${\text {end}}(Q)$ and ${\text {end}}(R)$ are directly connected, i.e. ${\text {end}}(Q) \cap {\text {end}}(R)\ne \emptyset$, or if there is an interval $v'$ among the intervals $I' \subseteq I \setminus P$, whose right endpoints ${\text {r}}(v') > {\text {r}}(v_{l})$ such that both ${\text {end}}(Q) \cap v' \ne \emptyset$ and ${\text {end}}(R) \cap v' \ne \emptyset$. In any of those two cases we can connect *Q* and ${\text {rev}}(R)$ to a Steiner cycle. Otherwise, the algorithm returns that no Steiner cycle exists.

### Theorem 4.1

The given algorithm correctly decides the existence of a Steiner cycle in *G*(*I*) and obtains such a cycle if possible.

### Proof

If the algorithm finds a Steiner cycle this is obviously true. Also, by correctness of the algorithm for the Steiner path cover (Theorem 3.3), if no Steiner path is found we correctly determine that no Steiner cycle can exist.

Otherwise, let us assume that the algorithm did not find a Steiner cycle. Without loss of generality, let ${\text {end}}(R) = v_{h}$ with $h < l-1$ and consider the path $P' = (v_{1}, v_{2}, \dots , v_{h})$ and its decomposition into covers and islands. Since *R* was not extended by any of the intervals $v_{h+2}, v_{h+3} \dots , v_{l}$, we have that $C(P') \cup \{v_{h+1}\}$ separates the islands of $P'$ from $\{v_{h+2}, v_{h+3}, \dots , v_{l}\}$. In addition, since ${\text {end}}(R)$ and ${\text {end}}(Q)$ could not be connected with any interval in $I'$, for all intervals $v' \in I'$ it holds that ${\text {l}}(v') > {\text {r}}(v_{h})$. Combining this with Proposition 3.1 we observe that $\{v_{h+2}, v_{h+3}, \dots , v_{l}\} \cup I'$ is non-empty and an *S*-island with respect to $C(P') \cup \{v_{h+1}\}$.

By Lemma 3.2 there are at least $|C(P')|+1$ *S*-islands with respect to $C(P') \cup \{v_{h+1}\}$. So in total we have at least $|C(P')|+2$ *S*-islands with respect to $C(P') \cup \{v_{h+1}\}$, hence by Proposition 2.2 there does not exist a Steiner cycle in *G*(*I*). $\square$

Given a Steiner path *P*, the paths *Q* and *R* can be easily constructed in *O*(*n*) time. This gives a linear time algorithm for the Steiner cycle problem in interval graphs.

**Fig. 2:**
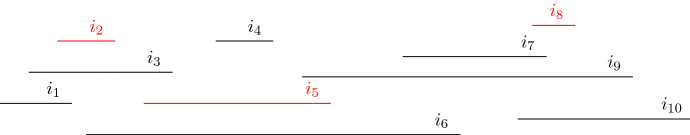
An instance of the Steiner cycle problem on an interval graph with $S=\{i_2, i_5, i_8\}$

Now we illustrate our algorithm for the Steiner cycle problem on interval graphs with the example given in Fig. 2. The given instance has 10 intervals $I=\{i_1, i_2, \ldots , i_{10}\}$ and $S=\{i_2, i_5, i_8\}$. Intervals in *S* are represented with the red color. First we run $\mathbf {GP}_S(I)$. It starts the path with $i_2$ and then extends it with $i_3$ and $i_5$ before neglecting $i_4$. Then it proceeds by extending the path with $i_6$, $i_7$, finishing with $i_8$. Hence it obtains the Steiner path $P = (i_2, i_3, i_5, i_6, i_7, i_8)$. In an attempt to create a Steiner cycle, we partition *P* into two paths *R* and *Q*. We initialize them with the first two intervals in *P*, that is, $R=(i_2)$ and $Q=(i_3)$. Now we consider *Q* to be the current path, and *R* to be the previous path. In each step we consider the next interval of *P*, and in the case that it intersect the end of the previous path, we extend the previous path and make it the current path. Otherwise we add the interval to the current path. So, interval $i_5$ is the next interval in *P*, and it does not intersect ${\text {end}}(R)= i_2$, hence we add it to *Q*, making it $Q=(i_3, i_5)$. The next interval is $i_6$, and it intersects ${\text {end}}(R)= i_2$, hence we extend *R* and make it the current path, so $R=(i_2, i_6)$. Next interval $i_7$ does not intersect ${\text {end}}(Q)= i_5$ so we extend *R* again, making it $R=(i_2,i_6, i_7)$. Finally, interval $i_8$ does not intersect ${\text {end}}(Q)= i_5$ so we extend *R*, making it $R=(i_2, i_6, i_7, i_8)$. This ends our partition of *P* with the resulting subpaths $R=(i_2, i_6, i_7, i_8)$ and $Q=(i_3, i_5)$. Since ${\text {end}}(R)=i_8$ and ${\text {end}}(Q)=i_5$ do not intersect, we cannot connect them into a cycle. The only remaining chance to do so is using an interval from $I' = \{i \in I\setminus P:{\text {r}}(i) > {\text {r}}({\text {end}}(P))\}= \{i_9, i_{10}\}$. Luckily, $i_9$ intersect both ${\text {end}}(R)=i_8$ and ${\text {end}}(Q)=i_5$, and can be used to connect *R* and *Q* into a cycle. The Steiner cycle is then given by $(R, i_9, {\text {rev}}(Q)) = (i_2, i_6, i_7, i_8, i_9, i_5, i_3)$.

Now let us consider a modified instance of Fig. 2, where $i_4$ is also an element of *S*. Then $\mathbf {GP}_S(I)$ would output the path $P=(i_2, i_3, i_5, i_4, i_6, i_7, i_8)$, and the subsequent partition of *P* would give $R=(i_2, i_6, i_7, i_8)$ and $Q=(i_3, i_5, i_4)$. But now there is no interval in $I'$ that connects ${\text {end}}(R)=i_8$ and ${\text {end}}(Q)=i_4$, so our algorithm outputs that there is no Steiner cycle. In order to verify that there is no Steiner cycle we can follow the arguments in the proof of Theorem 4.1, which gives us a cutset $C=\{i_5, i_6\}$ that separates *I* into three *S*-islands, and hence, by Proposition 2.2, guarantees that there is no Steiner cycle.

## Conclusion

We obtained linear time algorithms for both the Steiner path cover problem and the Steiner cycle problem on interval graphs, assuming the intervals are given as a endpoint sorted list.

It would be of interest to study these problems also for other types of intersection graphs, like for instance circular-arc graphs, for which efficient algorithms for the path cover problem and the hamiltonian cycle problem are known.
